# Enzymatic Synthesis of Lipophilic Esters of Phenolic Compounds, Evaluation of Their Antioxidant Activity and Effect on the Oxidative Stability of Selected Oils

**DOI:** 10.3390/biom11020314

**Published:** 2021-02-19

**Authors:** Bartłomiej Zieniuk, Katarzyna Groborz, Małgorzata Wołoszynowska, Katarzyna Ratusz, Ewa Białecka-Florjańczyk, Agata Fabiszewska

**Affiliations:** 1 Department of Chemistry, Institute of Food Sciences, Warsaw University of Life Sciences—SGGW, 159c Nowoursynowska St., 02-776 Warsaw, Poland; groborz.kasia@gmail.com (K.G.); ewa_bialecka_florjanczyk@sggw.edu.pl (E.B.-F.); agata_fabiszewska@sggw.edu.pl (A.F.); 2 Analytical Department, Łukasiewicz Research Network—Institute of Industrial Organic Chemistry, 6 Annopol St., 03-236 Warsaw, Poland; malgorzata.woloszynowska@ipo.lukasiewicz.gov.pl; 3 Department of Food Technology and Assessment, Institute of Food Sciences, Warsaw University of Life Sciences—SGGW, 159c Nowoursynowska St., 02-776 Warsaw, Poland; katarzyna_ratusz@sggw.edu.pl

**Keywords:** CALB, enzymatic esterification, phenolic compounds, antioxidant activity, oxidative stability, vanillyl hexanoate

## Abstract

The aim of the study was to compare the effect of the substituent and its position in the aromatic ring on the antioxidant activity of hexanoic acid esters obtained in reactions catalyzed by immobilized lipase B from *Candida antarctica*. 4-Hydroxybenzyl hexanoate, 2-hydroxybenzyl hexanoate, 4-methoxybenzyl hexanoate, and vanillyl hexanoate were obtained with conversion yields of 50 to 80%. The antioxidant activity of synthesized esters, their alcohol precursors and BHT (Butylated HydroxyToluene) was compared with DPPH (2,2-diphenyl-1-picrylhydrazyl), CUPRAC (cupric ion reducing antioxidant capacity), and CBA (crocin bleaching assay) methods. Furthermore, it was investigated whether the presence of vanillyl hexanoate in a concentration of 0.01 and 0.1% affected the oxidative stability of sunflower and rapeseed oils in the Rancimat test. It was observed that the antioxidant activity of hexanoic acid esters depends on the presence and position of the hydroxyl group in the aromatic ring. The highest activities were found for vanillyl alcohol, vanillyl hexanoate, and BHT. The addition of the ester and BHT significantly extended the induction times of the tested oils, and these compounds exhibited similar activity. Vanillyl hexanoate increased the induction time from 4.49 to 5.28 h and from 2.73 to 3.12 h in the case of rapeseed and sunflower oils, respectively.

## 1. Introduction

In recent years, the availability and consumption of highly processed and convenience food products has increased in many countries. Based on a Spanish study, for example, from 1990 to 2010, consumption of these food products increased threefold [1]. Many technological steps in the production chain and high processing of raw material can negatively affect the quality of the final product. Food additives are used in order to limit the adverse effects of food processing [2] and phenolic compounds are an interesting group of potential antioxidants.

Phenolic compounds are a large group of secondary metabolites that are diverse in structure and widespread in plants and mushrooms. The main and most widely distributed in nature classes of polyphenols are flavonoids, phenolic acids, stilbenes, and lignans [3]. The common part of these compounds is the presence of an aromatic ring with an attached hydroxyl group [4]. In addition to the previously mentioned classes, phenolics are a group containing about 5000 to 8000 forms of different structures, and also include such compounds as phenolic terpenes, alkylphenols, curcuminoids, and coumarins, along with phenolic derivatives of aldehydes, ketones, and alcohols [5]. The last aforementioned—aromatic alcohols—are a group of organic chemical compounds composed of an aromatic ring with an alkyl chain with a hydroxyl group attached, and the simplest example of such compounds is benzyl alcohol [6]. Aromatic alcohols, as well as other phenolic compounds, exhibit high biological activity, being antioxidant or antimicrobial agents and attracting the attention of many researchers due to the anticancer or neuro-, cardio- and hepatoprotective properties described in the literature [4]. Aromatic alcohols are very often used as fragrances and flavorants, and some of them, such as gastrodigenin (4-hydroxybenzyl alcohol), are found in plant extracts used in traditional medicine [7,8].

Biocatalysis provides opportunities for the sustainable production of chemical compounds with interesting features, which can possibly be commercialized as food additives [9,10]. Esterification of phenolic compounds is the primary method of their modification; moreover, the use of enzymes to catalyze these reactions has many advantages in comparison with conventional chemical synthesis. Enzyme-catalyzed reactions are carried out in milder conditions; fewer by-products are generated and they are environmental-friendly [10]. Different esters of phenolic compounds are used in the food or cosmetic industry and esterification improves their biological activity [4,9]. The approach has been presented in the current study that aimed to evaluate the effect of the substituent and its position in the aromatic ring on the antioxidant activity of hexanoic acid esters synthesized in reactions catalyzed by immobilized lipase B from *Candida antarctica* (CALB). Furthermore, it was investigated whether the presence of an ester characterized by the highest antioxidant activity in in vitro tests affected the oxidative stability of refined sunflower and rapeseed oils.

## 2. Materials and Methods

### 2.1. Materials

The biocatalyst used in this study was lipase B from *Candida antarctica* (CALB) immobilized on macroporous acrylic resin, which was purchased from Sigma-Aldrich (Poznan, Poland). Chemicals were purchased from Avantor Performance Materials Poland S.A. (Gliwice, Poland) and Sigma-Aldrich (Poznan, Poland). Refined vegetable oils—sunflower and rapeseed oils—were acquired from the local supermarket in Warsaw (Poland).

### 2.2. Esters Synthesis

**Figure 1:**
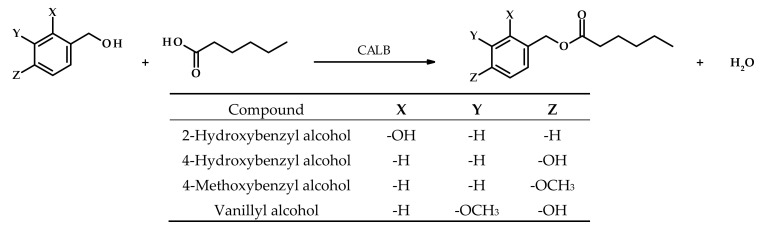
Lipase-catalyzed synthesis of hexanoic acid esters.

Syntheses of four esters were carried out with CALB as a biocatalyst (addition of 5% by weight of substrates). The esters were synthesized by reacting the appropriate aromatic alcohol—2-hydroxybenzyl, 4-hydroxybenzyl, 4-methoxybenzyl or 4-hydroxy-3-methoxybenzyl (vanillyl)—with hexanoic acid in a molar ratio of 1:2 (alcohol:acid) according to the reaction in Figure 1. Reactions were carried out in flasks in tert-butyl methyl ether at 37 °C at 200 rpm on a rotary shaker for 48 h.

### 2.3. Gas Chromatography

Samples were withdrawn from flasks after 1, 2, 24, and 48 h of reactions, and were subsequently derivatized with BSTFA (*N*,*O*-bis(trimethylsilyl)trifluoroacetamide) + 1% TMCS (trimethylchlorosilane) and pyridine added in equal volumes and heated for 30 min at 70 °C. Then, derivatized samples were analyzed by gas chromatography equipped with a flame ionization detector (GC-FID) using Agilent Technologies 7820A apparatus with an HP-5 column (0.25 mm, 30 m, 0.25 µm) (Agilent Technologies, Santa Clara, CA, USA). Nitrogen was used as a carrier gas at a flow rate of 1.5 mL/min. The temperature program of the GC analysis was as follows: 70 °C for 3 min, 70 to 150 °C (3 °C/min), 150 to 300 °C (40 °C/min), and 300 °C for 10 min. Injector and detector temperatures were 250 and 290 °C, respectively, with an injection volume of 1 µL. The percentage conversion was calculated based on the area under the peaks of aromatic alcohol and its ester.

### 2.4. Esters Purification

After 48 h, reactions were filtered through filter paper placed within the funnel to separate the enzyme. The solvent was evaporated in a vacuum evaporator (BUCHI Rotavapor R-200, Flawil, Switzerland) and the obtained mixtures were dissolved in 10 mL of chloroform. Then, 10 mL of a saturated solution of sodium bicarbonate was added to remove the excess of acid. Organic phases were collected and the solvent was evaporated. Prepared samples were applied to a silica gel column chromatography with a mixture of chloroform and methanol (9:1, v/v) as an eluent.

Structures of the synthesized esters were confirmed after their purification using ^1^H NMR. Compound spectra were recorded using a Bruker AVANCE 300 MHz spectrometer (Bruker, Billerica, MA, USA) with CDCl_3_ as a solvent. Proton chemical shifts of obtained esters are reported below in ppm (δ) relative to tetramethylsilane (TMS), which was used as an internal standard.

**2-Hydroxybenzyl hexanoate**^1^H–NMR (300 MHz, CDCl_3_): δ 0.87 (3H, t, *J* = 7.1 Hz), 1.20–1.42 (4H, m), 1.66 (2H, m), 2.37 (2H, t, *J* = 7.1 Hz), 5.15 (2H, s), 6.87–7.02 (2H, m), 7.24–7.36 (2H, m), 7.87 (1H, s)

**4-Hydroxybenzyl hexanoate**^1^H–NMR (300 MHz, CDCl_3_): δ 0.87 (3H, t, *J* = 7.1 Hz), 1.28–1.31 (4H, m), 1.64 (2H, m), 2.34 (2H, t, *J* = 7.1 Hz), 5.05 (2H, s), 5.85 (1H, s), 6.81–6.83 (2H, m), 7.22–7.24 (2H, m)

**4-Methoxybenzyl hexanoate**^1^H–NMR (300 MHz, CDCl_3_): δ 0.88 (3H, t, *J* = 7.1 Hz), 1.25–1.42 (4H, m), 1.63 (2H, m), 2.36 (2H, t, *J* = 7.1 Hz), 3.83 (3H, s), 5.07 (2H, s), 6.86–6.97 (2H, m), 7.26–7.37 (2H, m)

**Vanillyl hexanoate**^1^H–NMR (300 MHz, CDCl_3_): δ 0.88 (3H, t, *J* = 7.1 Hz), 1.25–1.36 (4H, m), 1.66 (2H, m), 2.35 (2H, t, *J* = 7.1 Hz), 3.92 (3H, s), 5.05 (2H, s), 5.66 (1H, s), 6.85–6.97 (3H, m)

### 2.5. Evaluation of Antioxidant Activity

The following methods for the determination of the antioxidant activity of tested compounds were applied: the method with DPPH• radical, the CUPRAC method and crocin bleaching assay (CBA).

The DPPH• (2,2-Diphenyl-1-picrylhydrazyl) radical scavenging method was used according to the previously reported protocol by Zanetti et al. [11]. The IC_50_ parameter, i.e., the concentration required for a 50% reduction of the DPPH• radical—was calculated for all esters and their precursors.

The principle of the CUPRAC (cupric ion reducing antioxidant capacity) method is a spectrophotometric measurement of the absorption of the formed complex of a Cu (I) ion with neocuproine (2,9-dimethyl-1,10-phenanthroline). According to Özyürek et al. [12], the Trolox equivalent antioxidant capacities (TEAC) were determined for the tested compounds based on the absorbance of compounds and Trolox, used as a reference standard.

Compounds were characterized by the highest antioxidant activity in the DPPH• radical scavenging method and CUPRAC method; i.e., vanillyl alcohol and vanillyl hexanoate were used in crocin bleaching assay. CBA is an in vitro method for measuring the antioxidant capacity of different samples (pharmaceuticals, pure compounds or plant extracts). This method is based on the spectrophotometric measurement of the inhibition of crocin discoloration by peroxide radicals in the presence of antioxidants. The protocol by Ordoudi and Tsimidou [13] was used with AAPH (2,2′-Azobis(2-methylpropionamidine) dihydrochloride), a water-soluble azo-initiator that is responsible for the generation of peroxyl radicals after its thermolysis.

In all of the above-mentioned methods, the known commercial synthetic antioxidant—Butylated HydroxyToluene (BHT)—was used as a reference compound.

### 2.6. Determination of Oils Quality Parameters

The acid and peroxide values (AVs and PVs, respectively) were determined in refined vegetable oils used in the current study. The AV was determined according to ISO 660:2009 and the results were expressed as mg KOH/g of oil. The PV was determined with iodometric titration according to ISO 3960:2017 and expressed as mEq O_2_/kg of oil.

### 2.7. Determination of the Fatty Acid Composition of Vegetable Oils

The fatty acid compositions of rapeseed and sunflower oils were determined after derivatization to methyl esters and analyzed by gas chromatography equipped with a flame ionization detector (GC-FID) using Agilent Technologies 7820A with a ZB-FFAP capillary column (30 m, 0.25 mm, 0.25 μm) (Agilent Technologies, Santa Clara, CA, USA) with helium as the carrier gas. The temperature program of the separation of FAME (fatty acid methyl esters) was as follows: 120 °C for 2 min, 120 to 200 °C (10 °C/min), 200 °C for 5 min, 200 to 240 °C (3 °C/min), and then 240 °C for 10 min. The injector and detector temperatures were 260 and 280 °C, respectively, with an injection volume of 1 µL. Fatty acids were identified based on the comparison of their retention times with those of analytical standards.

### 2.8. Oxidative Stability Measurements Using the Rancimat Method

To determine the oxidative stability of the oils, 743 Rancimat apparatus (Metrohm, Herisau, Switzerland) with the following conditions were used: 2.5 g of sample, constant air flow of 20 L/h, and a temperature of 120 °C [14,15]. The experiment examined the effect of vanillyl hexanoate in two different concentrations (0.01% and 0.1%) on the oxidative stability of refined oils. BHT at a concentration of 0.01% was used as a control antioxidant compound.

### 2.9. Statistical Analysis

Statistical analysis was performed using Statistica 13.3 software (TIBCO Software Inc., Palo Alto, CA, USA). The results were analyzed using one-way analysis of variance (ANOVA) and Tukey’s post-hoc test. The significance level was α = 0.05.

## 3. Results and Discussion

### 3.1. Enzymatic Esterification

**Figure 2:**
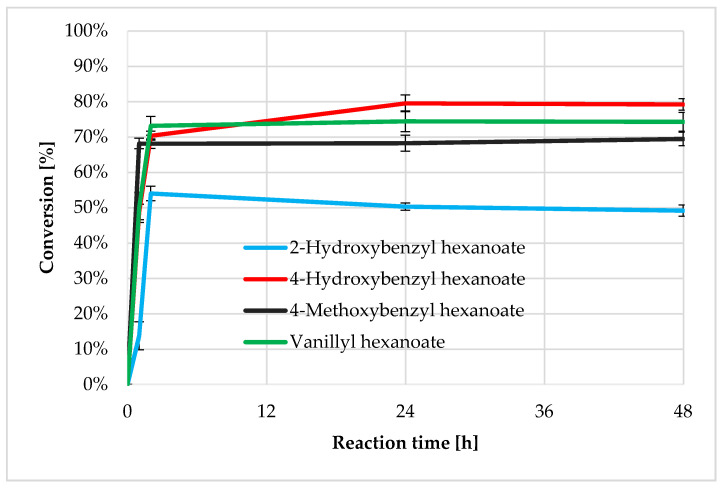
Percentage conversion (% ± SD) of aromatic alcohols and hexanoic acid to their esters with CALB after 1, 2, 24, and 48 h of reaction

As a result of the esterification reactions of hexanoic acid with aromatic alcohols, the following four esters were obtained: 4-hydroxybenzyl hexanoate, 2-hydroxybenzyl hexanoate, 4-methoxybenzyl hexanoate, and vanillyl hexanoate. The progress of the reactions carried out in the current work was monitored after 1, 2, 24, and 48 h of reaction by gas chromatography; the conversions are presented in Figure 2. All reactions catalyzed by lipase B from *C. antarctica* were characterized by a high (over 50%) degree of reacting of the substrates after two hours of reaction. Substrate conversion after 2 h was between 54 and 74%; up to 48 h, these values changed slightly and were 50 to 80%. The synthesis of 4-hydroxybenzyl hexanoate was characterized by the highest conversion (about 80% after 24 h). The reaction for which the lowest degree of conversion was observed was the synthesis of 2-hydroxybenzyl hexanoate. The reaction equilibrium was established at about 50% conversion. It was observed that the presence of the hydroxyl group in the ortho- position in the 2-hydroxybenzyl alcohol molecule negatively affected the esterification reaction compared to the reaction with 4-hydroxybenzyl alcohol (with the hydroxyl group in the para- position). The observed relationship is probably related to the substrate specificity of the enzyme used as well as substrate access to the active site of the enzyme.

Fuentes et al. [16] studied the relationship between lipase structure and its catalytic activity. Molecular modelling studies compared two lipases of microbial origin—*C. antarctica* and *Thermomyces lanuginosus*—investigating possible transition states for the sucrose and vinyl laurate transesterification reaction. Both enzymes showed a broad ligand binding spectrum in the substrate-binding pocket, while lipase B from *C. antarctica* had a wider pocket size and hence showed less selectivity for reaction products.

In addition, Otto et al. [17] explained the potential and limitations of CALB in the synthesis of aromatic glycolipids. The enzymatic reaction has been found to be influenced by: (a) The chain length of acyl donors; at least one methylene bridge should be present between the carboxylic group and the aromatic ring; (b) The substitution pattern of the aromatic ring; the hydroxyl group in the ortho- position negatively affected the reaction yield compared to the groups in the para or meta positions [17].

The above-mentioned studies confirmed that the catalyst used in this study is a versatile enzyme, but still with some limitations. Nevertheless, the CALB enzyme is capable of catalyzing the esterification reaction of some atypical carboxylic acids and alcohols.

### 3.2. Antioxidant Activity of Obtained Esters

The obtained compounds, as well as their precursors, were tested for antioxidant properties by means of two methods: CUPRAC and DPPH (Table 1). It has been shown that the tested compounds significantly differed in the values of their antioxidant activity assessed by the CUPRAC method. The Trolox equivalent antioxidant capacity (TEAC) values ranged between 0.026 (for 4-methoxybenzyl hexanoate) and 1.086 (for vanillyl alcohol). In addition to vanillyl alcohol, a high TEAC value was also found for vanillyl hexanoate (0.889). These compounds were characterized by higher antioxidant activity than the industrially used antioxidant BHT (0.739). The antioxidant activity measured using the DPPH radical method and the CUPRAC method were comparable. According to the principle of the DPPH method, the lower the IC_50_ value (indicating the concentration at which half of the DPPH radicals are scavenged), the higher the antioxidant activity. The values obtained ranged between 0.28 (vanillyl alcohol) and 864.78 mM (4-methoxybenzyl hexanoate). Outstanding antioxidant values were found also for vanillyl hexanoate (1.71) and BHT (0.47).

The structure of aromatic alcohols used in the ester synthesis reactions significantly affected the antioxidant properties of the obtained compounds. It was found that the antioxidant activity of hexanoic acid esters depends on the presence and position of the hydroxyl group in the aromatic ring. Ester, as well as its precursor with a hydroxyl group in the ortho- position, showed higher antioxidant activity in the CUPRAC method than ester with a hydroxyl group in the para- position (TEAC = 0.407 for 2-hydroxybenzyl hexanoate, and TEAC = 0.294 for 4-hydroxybenzyl hexanoate). On the other hand, the presence of a methoxy group in the aromatic ring did not affect the antioxidant activity (IC_50_ = 864.78 mM and TEAC = 0.026 for 4-methoxybenzyl hexanoate), but it has been noticed that this group had an enhancing effect on the hydroxyl group; hence, the presence of a hydroxyl group in the para- position and a methoxy group in the meta- position relative to the carbon chain in the aromatic ring predisposed vanillyl alcohol and its derivatives towards increased antioxidant properties.

The results obtained in the current study, as well as in study by Noriega-Iribe et al. [18], confirmed that the presence of electron-donating groups such as hydroxy and *p*-dimethylamino groups in the aromatic ring are relevant for the antioxidant activity of chemical compounds. In the research by Dhiman et al. [19], similar observations regarding the position of the hydroxyl group in hydroxybenzyl alcohols have been noted. 2-Hydroxybenzyl alcohol proved to be a better antioxidant compared with 4-hydroxybenzyl alcohol and was a better ABTS radical scavenger than the 3-hydroxybenzyl and 4-hydroxybenzyl alcohols [19]. Velika and Kron [20] also confronted the position of the hydroxyl group in hydroxybenzoic acids. 2-Hydroxybenzoic acid was found to be a stronger antioxidant than 4-hydroxybenzoic acid and was far better than 3-hydroxybenzoic acid [20].

The introduction of additional electron-donating groups to the phenolic ring enhances the antioxidant activity. Intramolecular hydrogen bonding of two substituted phenolic compounds contributed to the stabilization of the phenoxyl radical formed after hydrogen donation [21]. Farhoosh et al. [22] acknowledged that an extra hydroxyl group was a more powerful electron donor in comparison with methoxy group, which significantly increased the antioxidant properties of the tested compounds.

Natalia et al. [23] and Park et al. [24] synthesized enzymatically fatty acid vanillyl esters with menhaden and castor oils, respectively. The DPPH radical scavenging activity of the tested compounds strongly depends on the solvent used in the test. Fatty acid vanillyl esters showed similar IC_50_ values in 1-butanol and toluene, unlike their precursor, vanillyl alcohol, where its activity decreased significantly in the more non-polar solvent [23]. The same observations were noticed by Park et al. [24] and ricinoleic acid vanillyl ester exhibited comparable antioxidant activity in methanol, 2-propanol, 1-butanol, and toluene. In the current study, the water–ethanol environment in the CUPRAC method and methanol as a solvent in the DPPH radical method were used. The use of polar solvents may be the reason that the obtained esters had a lower antioxidant potency than their precursors. Both Roleira et al. [25] and Gaspar et al. [26] concluded that, in general, phenolic compounds such as ferulic, caffeic, and sinapic acids have higher antioxidant activity than their ester or amide derivatives, but esterification positively affects the lipophilicity of the compounds obtained, which increases their utility as antioxidant agents in lipid-rich media.

### 3.3. Evaluation of Antioxidant Activity by Means of Crocin Bleaching Assay

Vanillyl hexanoate was selected for further analysis alongside its precursor—vanillyl alcohol—and BHT was tested by crocin bleaching assay. Results of the comparison are presented in Table 2 in three ways: percentage inhibitory effect, Trolox equivalent values (TEVs), and IC_50_. Compounds showed similar activity, and vanillyl alcohol had the best inhibitory effect of peroxyl radicals, which was about 46%. The rest of the compounds exhibited slightly lower values—39% for vanillyl hexanoate and 34% for BHT. In the case of TEV, vanillyl alcohol and its hexanoic ester obtained values were 0.50 and 0.42, respectively.

Most of the CBA applications relate to hydrophilic radical scavengers [27]. Vanillyl alcohol is definitely a better soluble in hydrophilic media than its ester. CBA is a method involving hydrogen-atom transfer (HAT) in contrast to single-electron transfer (SET) methods like DPPH and CUPRAC assays. Nevertheless, the presence, quantity, and arrangement of electron-donating groups are the most important factors determining high antioxidant activity. Ordoudi and Tsimidou [13] investigated 39 phenolic compounds by the CBA method. Single phenols were more active than their respective hydroxybenzoic acids. Pyrogallol and gallic acid proved to be the best scavengers of the peroxyl radicals, and the effect of proximity of the carboxylic group to the aromatic ring also affected the compound activity.

### 3.4. The Effect of Vanillyl Hexanoate on the Oxidative Stability of Rapeseed and Sunflower Oils

The choice of antioxidant activity testing methods is often burdened with some disadvantages. It should be taken into account that these methods show some specificity in terms of mechanism of action, pH, temperature, time, etc. In addition, various compounds are used as standards for the construction of calibration curves, such as Trolox, gallic, chlorogenic or ascorbic acids. Therefore, in vitro methods can not accurately reflect the antioxidant capacity of tested compounds [28,29]. In fact, the activity of antioxidants not only depends on the structure, but also on the concentration, temperature, and type of substrate, including the presence of prooxidants or synergists, or the system into which the compound is introduced [30].

The esterification of phenolic compounds for the synthesis of molecules with medium or long aliphatic chains can be used as a method for the modification of physical properties, e.g., solubility, miscibility, and antioxidant activity in emulsion-based systems [31]. Therefore, in the current study, it was verified whether the ester that had the best antioxidant properties described by in vitro methods would be able to affect the oxidative stability of commonly consumed vegetable oils. Oils are food products susceptible to the oxidation processes in the presence of factors such as high temperature, light, enzymes or the presence of microorganisms. The oxidative stability of the oil is a very important quality feature and it determines the technological usefulness of a given product [30].

The acid and peroxide values as well as the fatty acid profiles of rapeseed and sunflower oils are shown in Table 3. These quality features are a measure of free fatty acids and the extent to which an oil has undergone primary oxidation, respectively. The obtained acid values were as follows: 0.22 mg KOH/g for rapeseed oil and 0.33 mg KOH/g for sunflower oil. Moreover, plant oils were characterized by peroxide values of 1.79 mEq O_2_/kg and 2.20 mEq O_2_/kg, respectively. The tested oils were of good quality, meeting the requirements of PN-A-86908:2000, where, in the case of PV, it should not be higher than 5 mEq O_2_/kg, and the rate of fat hydrolysis (AV) not higher than 0.3 mg KOH/g. Similarly, the fatty acid composition of the tested oils met the requirements of Codex Alimentarius standards [32].

The oxidation induction times of rapeseed and sunflower oils with or without the addition of vanillyl hexanoate (0.01% or 0.1%) or BHT (0.01%) are shown in Table 4. Rapeseed oil proved to be more stable (induction time = 4.49 h) than sunflower oil (induction time = 2.73 h), due to the lower content of polyunsaturated fatty acids. The latter is characterized by a high content of linoleic acid (60.9%), which is more susceptible to oxidation. The addition of ester and BHT significantly extended the induction time of the tested oils. Based on the induction times, protection factors were calculated (Table 4). Values greater than 1 confirm the beneficial effect of the used antioxidants on the oxidative stability of the oils. Vanillyl hexanoate in the concentration of 0.1% exhibited similar activity to BHT and increased the induction time from 4.49 h to 5.28 h and from 2.73 h to 3.12 h in the case of rapeseed and sunflower oils, respectively. In the case of the first oil, the statistical significance of using a higher concentration of vanillyl hexanoate is also noticeable with oxidation induction times of: 5.28 h and 4.90 h, as well as protection factors of 1.18 and 1.09.

The enzymatic synthesis of lipophilic esters as antioxidants for oil stability application was investigated by Buisman et al. [31] and Viklund et al. [33]. The first research group used CALB to obtain octanoate esters of benzyl alcohol derivatives. Synthesized 3,4-dihydroxyphenethyl and 3,5-di-*tert*-butyl-4-hydroxybenzyl octanoates significantly increased the induction time of sunflower oil [31]. In the case of Viklund et al. [33], enzymatically obtained palmitate and oleate esters of ascorbic acid showed antioxidant activity, which was noticed by the lower values of PV in accelerated storage tests of rapeseed oil incubated with ascorbyl fatty acid esters compared to the oil without addition.

The subject of using esters of phenolic compounds as lipophilic antioxidants was also explored by Sørensen et al. [34,35]. In acid-catalyzed reactions, they obtained methyl, butyl, octyl, dodecyl, hexadecyl, and eicosyl esters of ferulic [34] and caffeic acids [35], respectively. Alkyl chain length in tested ferulates influenced fish oil-enriched milk oxidative stability. Interestingly, the most efficient compound proved to be methyl ferulate, followed by ferulic acid and its butyl ester. Elongation of the alkyl chain, in the case of dodecyl and octyl ferulates, caused counterproductive effects and prooxidative effects were observed [34]. Similar conclusions were also reported with the use of caffeic acid esters in O/W emulsions. They have shown that antioxidant efficiency was dependent on the presence of endogenous tocopherol, the use of various emulsifiers, and the partitioning of the antioxidants in different phases, as well as interactions between the aforementioned factors [35].

## 4. Conclusions

Looking at the problems faced by the modern world, including the food industry, biotransformation processes can be an interesting alternative to traditional chemical synthesis. The current work presents the synthesis of aromatic alcohol esters by biocatalysis with lipase B from *C. antarctica*. It was observed that the antioxidant activity of hexanoic acid esters depends on the presence and position of the hydroxyl group in the aromatic ring. One of the synthesized compounds—vanillyl hexanoate—could potentially be used as a food additive with antioxidant properties. The addition of the mentioned ester significantly extended the induction time of the tested oils from 4.49 to 5.28 h and from 2.73 to 3.12 h in the case of rapeseed and sunflower oils, respectively. Phenolic compounds have known antioxidant properties, but are characterized by low solubility in lipid matrices. Esterification aimed at increasing their lipophilicity without losing their ability to neutralize free radicals. It is worth continuing work on the use of the obtained esters to increase the oxidative stability of food products subjected to various technological treatments or during storage.

## Figures and Tables

**Table 1:** Comparison of antioxidant activity of tested compounds by means of methods with DPPH• radical and CUPRAC.

Compound	Antioxidant Activity	
	DPPH•	CUPRAC
	IC_50_ (mM) *	TEAC **
Vanillyl alcohol	0.28 ± 0.01 ^A^ ***	1.086 ± 0.025 ^A^
2-Hydroxybenzyl alcohol	107.37 ± 2.47 ^B^	0.452 ± 0.004 ^D^
4-Hydroxybenzyl alcohol	126.81 ± 9.53 ^B^	0.338 ± 0.025 ^E^
4-Methoxybenzyl alcohol	388.00 ± 34.98 ^C^	0.032 ± 0.005 ^F^
Vanillyl hexanoate	1.71 ± 0.07 ^A^	0.889 ± 0.025 ^B^
2-Hydroxybenzyl hexanoate	128.51 ± 2.78 ^B^	0.407 ± 0.008 ^D^
4-Hydroxybenzyl hexanoate	137.32 ± 5.06 ^B^	0.294 ± 0.007 ^E^
4-Methoxybenzyl hexanoate	864.78 ± 56.32 ^D^	0.026 ± 0.001 ^F^
Butylated hydroxytoluene (BHT)	0.47 ± 0.08 ^A^	0.739 ± 0.048 ^C^

* Concentration required for 50% reduction of the DPPH• radical, ** Trolox equivalent antioxidant capacity, *** The values with the same letter in a column did not differ significantly (*α* = 0.05).

**Table 2:** Comparison of antioxidant activity of vanillin alcohol, vanillyl hexanoate, and BHT by crocin bleaching assay.

Compound	Crocin Bleaching Assay		
	%_INH_ * (%)	TEV **	IC_50_ *** (µM)
Vanillyl alcohol	46 ± 2 ^A^	0.50 ± 0.02 ^A^	107 ± 3 ^A^
Vanillyl hexanoate	39 ± 3 ^B^	0.42 ± 0.03 ^B^	128 ± 6 ^B^
Butylated hydroxytoluene (BHT)	34 ± 1 ^B^	0.37 ± 0.01 ^B^	137 ± 4 ^B^

* Percentage inhibitory effect, ** Trolox Equivalent Values, *** Concentration corresponding to 50% inhibitory effect; The values with the same letter in a column did not differ significantly (*α* = 0.05).

**Table 3:** Acid and peroxide values, and fatty acid profiles of rapeseed and sunflower oils.

Parameter	Rapeseed Oil	Sunflower Oil	According to PN-A-86908:2000	
Acid value (AV) [mg KOH/g]	0.22 ± 0.004 ^A^ *	0.33 ± 0.004 ^B^	≤0.3	
Peroxide value (PV) [mEq O_2_/kg]	1.79 ± 0.010 ^A^	2.20 ± 0.043 ^B^	≤5.0	
**Fatty acid composition (%)**			**According** **to Codex Alimentarius** [32]	
Fatty Acid name	Rapeseed oil	Sunflower oil	Rapeseed oil (low erucic acid)	Sunflower oil
Palmitic (C16:0)	4.9	5.7	2.5–7.0	5.0–7.6
Stearic (C18:0)	1.6	3.2	0.8–3.0	2.7–6.5
Oleic (C18:1)	66.4	25.4	51.0–70.0	14.0–39.4
Linoleic (C18:2)	17.1	60.9	15.0–30.0	48.3–74.0
Linolenic (C18:3)	6.5	0.2	5.0–14.0	ND–0.3
Arachidic (C20:0)	0.6	0.4	0.2–1.2	0.1–0.5
Eicosenoic (C20:1)	1.5	0.2	0.1–4.3	ND–0.3
Behenic (C22:0)	0.3	0.6	ND**–0.6	0.3–1.5
Erucic (C22:1)	0.7	0.1	ND–2.0	ND–0.3
∑ Saturated Fatty Acids ∑ (SFA)	7.4	9.9	3.5–11.8	8.1–16.1
∑ Monounsaturated Fatty Acids ∑ (MUFA)	68.6	25.7	51.1–76.3	14.0–40.0
∑ Polyunsaturated Fatty Acids ∑ (PUFA)	23.6	61.1	20.0–44.0	48.3–74.3

* The values with the same letter in a row did not differ significantly (*α* = 0.05); ** ND–Not Detected.

**Table 4:** Oxidation induction times [h] for rapeseed and sunflower oils with or without addition of vanillyl hexanoate (0.01% or 0.1%) or BHT (0.01%) and calculated protection factors.

	Rapeseed Oil		Sunflower Oil	
	Induction Time (h)	Protection Factor	Induction Time (h)	Protection Factor
Control	4.49 ± 0.08 ^A^ *	-	2.73 ± 0.08 ^A^	-
Vanillyl hexanoate 0.01%	4.90 ± 0.01 ^B^	1.09	2.89 ± 0.09 ^AB^	1.06
Vanillyl hexanoate 0.1%	5.28 ± 0.17 ^C^	1.18	3.12 ± 0.06 ^B^	1.14
BHT 0.01%	5.22 ± 0.07 ^C^	1.16	3.10 ± 0.09 ^B^	1.14

* The values with the same letter in a column did not differ significantly (α = 0.05)

## Data Availability

The data presented in this study are available on request from the corresponding author (B.Z.).
